# Dietary Supplementation for Para-Athletes: A Systematic Review

**DOI:** 10.3390/nu13062016

**Published:** 2021-06-11

**Authors:** Keely A. Shaw, Gordon A. Zello, Brian Bandy, Jongbum Ko, Leandy Bertrand, Philip D. Chilibeck

**Affiliations:** 1 College of Kinesiology, University of Saskatchewan, Saskatoon, SK S7N 5B2, Canada; jongbum.ko@usask.ca (J.K.); ler149@usask.ca (L.B.); phil.chilibeck@usask.ca (P.D.C.); 2 College of Pharmacy and Nutrition, University of Saskatchewan, Saskatoon, SK S7N 5E5, Canada; gordon.zello@usask.ca (G.A.Z.); b.bandy@usask.ca (B.B.)

**Keywords:** paralympics, sport nutrition, caffeine, creatine, spinal cord injury, brain injury, cerebral palsy

## Abstract

The use of dietary supplements is high among athletes and non-athletes alike, as well as able-bodied individuals and those with impairments. However, evidence is lacking in the use of dietary supplements for sport performance in a para-athlete population (e.g., those training for the Paralympics or similar competition). Our objective was to examine the literature regarding evidence for various sport supplements in a para-athlete population. A comprehensive literature search was conducted using PubMed, SPORTDiscus, MedLine, and Rehabilitation and Sports Medicine Source. Fifteen studies met our inclusion criteria and were included in our review. Seven varieties of supplements were investigated in the studies reviewed, including caffeine, creatine, buffering agents, fish oil, leucine, and vitamin D. The evidence for each of these supplements remains inconclusive, with varying results between studies. Limitations of research in this area include the heterogeneity of the subjects within the population regarding functionality and impairment. Very few studies included individuals with impairments other than spinal cord injury. Overall, more research is needed to strengthen the evidence for or against supplement use in para-athletes. Future research is also recommended on performance in para-athlete populations with classifiable impairments other than spinal cord injuries.

## 1. Introduction

Over half of the US population uses dietary supplements [1]. Athletes tend to consume even higher amounts with up to 75% of athletes reporting the use of dietary supplements [2]. Athletes take supplements for various reasons, including enhanced sport performance, overall health and well-being, combatting jet lag, increased energy, and others [3]. Recent literature suggests that the prevalence of supplement use in athletes with a disability (i.e., para-athletes) is also high [4].

A dietary supplement is defined as “a food, food component, nutrient, or non-food compound that is purposefully ingested in addition to the habitually-consumed diet with the aim of achieving specific health and/or performance benefits” [3]. Extensive research has been carried out in able-bodied (AB) athletes investigating the performance-enhancing potential for various supplements; however, the literature investigating the same question in the para-athlete population is lacking. Recommendations based on evidence from able-bodied athletes are most likely inappropriate for a para-athlete group due to alterations in physiologic and metabolic responses, as well as potential implications of each individual’s specific impairment [5]. To date, a single systematic review exists for dietary supplements for performance in a para-athlete group, focusing solely on a spinal cord injury (SCI) population [6]. Given that para sport involves a variety of conditions, SCI being only one, it is important to understand how different supplements impact the performance outcomes of other para-athletes such as those with amputations, neurological conditions, etc.

Due to the heterogeneity within para-athletes, nutritional and supplement advice is likely to vary significantly on an individual basis and should take into account both health and performance [7]. When considering a para-athlete group, one must consider the following potential implications: altered metabolic rate and energy expenditure, reduced muscle mass, drug-nutrient interactions with medications, difficulty swallowing, altered thermoregulation, and others [8]. Such implications may change the athlete’s energy demands, the type of foods that can be consumed, and the potential for differing levels of effectiveness of supplements in a para-athlete group relative to their AB peers. Further, the pharmacokinetics of some drugs/supplements might differ in spinal cord injury, or side effects such as tremors from caffeine or gastrointestinal discomfort from creatine may impact those with impairments so that a performance benefit is no longer evident. Similarly, supplements that do not have a strong level of evidence in an AB population might have a greater performance impact in those with impairments due to differences in physiology.

Currently, nutritional guidelines including the efficacy of supplements for athletes with impairments have not been developed [4]. This is due to a lack of evidence on the benefits of supplements for sport performance in para-athlete groups aside from SCI. Due to differing physiology in individuals with physical impairments, evidence for supplements in AB athletes cannot be directly applied to athletes with impairments. Therefore, the purpose of this review is to examine the current status of literature concerning best practices of sport supplementation for the para-athlete, inclusive of various conditions such as brain injury and other neurological conditions, multiple sclerosis, SCI, muscular dystrophy, and limb deficiency.

### Background

The Paralympic Games are the epitome of sporting events for those with physical and visual impairment. These Games have had an immense impact on changing society’s perception of disabilities and emphasizing achievement rather than impairment [9]. The diversity of athletes taking part in such sport is vast, with a taxonomy designed to provide an evidence-based approach to categorize individual athletes of similar levels of function together to ensure fair competition in a relatively heterogenous group [10]. Athletes are classified based on measurable characteristics [11] such as visual impairment, limb deficiency, impaired muscle power and range of motion, short stature, hypertonia, ataxia, and athetosis [12]. These impairments are then assessed based on the impact on the fundamental activities of each Paralympic sport [10]. This diversity of impairment leads to a vast heterogeneity amongst athletes in the same sport. For example, in the sport of paracycling, athletes are classified into categories based on what type of bike they ride (B- tandem; C- a regular, two-wheeled bike; H- handbike; T- tricycle) and subsequently into sub-categories for the C, T, and H classes based on their level of impairment (C1-5, H1-5, T1-2) with a lower number corresponding with a greater degree of impairment. A C5 athlete might have an upper limb amputation or malformity at the wrist. In contrast, a C1 athlete might have a lower limb deficiency at the hip and impairment in the upper body. Likewise, an H5 athlete often has a double leg deficiency above the knee, while an H1 athlete would have tetraplegia with impairments corresponding to a motor complete cervical lesion at C6 or above [13]. Some sports governed by the International Paralympic Committee also include or are exclusive to those with visual impairment but no physical impairment. Due to the inherent differences in physical abilities in this group of athletes, specific dietary recommendations need to be developed for the different levels of impairment which may include the use of supplements. Those with visual impairment or minor physical impairment (i.e., finger/hand amputation) can likely derive their dietary and supplementation recommendations from the AB literature due to the minimal physiological or metabolic implications. However, for those with more severe physical limitations, specific recommendations should be considered and applied.

## 2. Methods

Our systematic review investigating the use of dietary supplements as an ergogenic aid was completed as per the Preferred Reporting Items for Systematic Review and Meta-Analysis (PRISMA) statement [14] and was registered with PROSPERO (CRD42020210762) on October 23, 2020. A literature search was conducted using the databases PubMed, SPORTDiscus, MedLine, and Rehabilitation and Sports Medicine Source, including all dates up to 3 May 2021. The following keywords and Boolean phrases were used: (para OR Paralympic OR athlete with disabilities OR cerebral palsy OR brain injury OR wheelchair OR handcycling OR hand OR amputee OR amputation OR limb deficiency) AND (nutritional supplement OR dietary supplement OR caffeine OR creatine OR beta-alanine OR buffer OR vitamin OR leucine OR lysine OR glutamine OR beta-hydroxy beta-methylbutyric acid OR arginine OR amino acid OR beet OR beetroot OR nitrate OR tea OR green tea OR matcha OR matcha tea OR CBD OR cannabinoid OR protein). A restriction was placed to include only human trials, but no restrictions were placed in terms of language or date.

The following population, intervention, comparator, outcomes, and study types (PICOS) were included: The population was individuals with Paralympic classifiable physical impairments [15,16]. The intervention was any variety of dietary supplementation, acute or chronic. Studies with any variety of comparators were considered (placebo, no supplement, no comparators). The outcome measures were any variety of exercise measures (i.e., aerobic power, aerobic capacity, time trial, strength, etc.). Due to the limited amount of research in the area, we considered all study types. Only published material was considered. Risk of bias for selected studies was determined by two researchers using the Cochrane risk of bias tool [17], using additional considerations for cross-over trials [18]. Titles, abstracts, and manuscripts were also reviewed by two researchers to determine eligibility based on the outlined PICOS parameters. Any disagreements were settled by a third reviewer.

## 3. Results and Discussion

**Figure 1:**
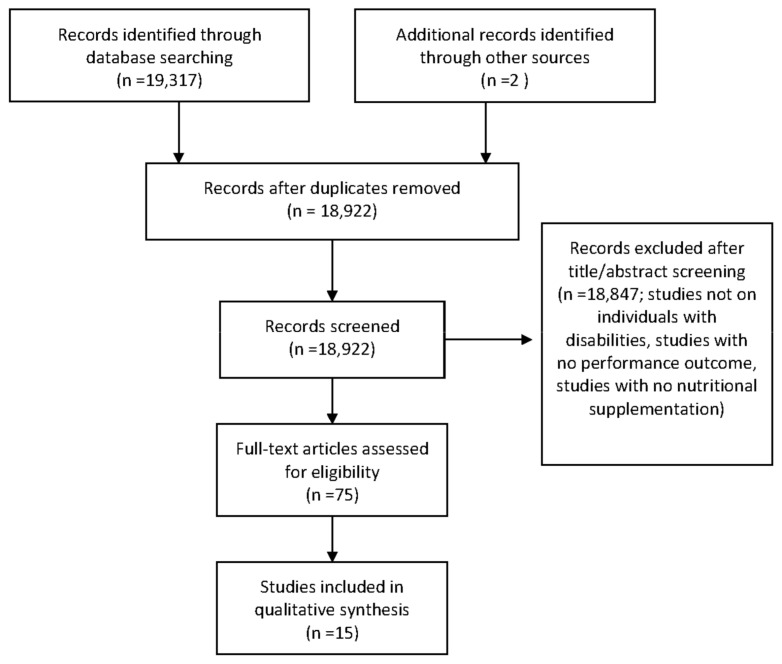
PRISMA diagram: Flow chart of study section process.

A detailed outline of the study selection process is presented in Figure 1. A total of 15 articles involving 311 participants were found to meet all criteria to be included in the review. Of these, 13 articles were clinical trials, [19,20,21,22,23,24,25,26,27,28,29,30,31], one was a case study [32], and one was a single-arm design [33]. The articles examined populations such as SCI [6,19,20,21,22,23,24,25,26,28,30,32,33], spina bifida [33], neurological conditions [29], muscular dystrophy [27], cerebral palsy [21,31] and cauda equina [33]. 

Supplements investigated included caffeine [22,23,24,25,32], creatine [19,26,27,28], fish oil [30], nitrates [20], vitamin D [19,21,33], leucine [31], and sodium citrate [23]. Six studies involved participants considered as elite athletes [20,21,23,24,25,33], four were described as athletes but the level at which they competed was not reported [22,24,26,30], while the activity level/athlete status was not reported in four studies [19,27,28,29]. An overview of population, supplement, study design, and outcomes can be found in Table 1. 

### 3.1. Risk of Bias

Of the 15 articles included, three were considered low risk of bias, eight had some concerns, and three had high risk (Table 2). One [32] was a case study and could not be assessed using the tool which was designed for trials. However, given the type of study, it was deemed high risk. For the randomization of included studies, some [21,30,33] were not randomized and, therefore, considered to have a high risk of bias. Most studies deemed to have “some concerns” with selection of reported results were categorized as such because whether the trial was analyzed with a pre-specified plan could not be determined (i.e., study was not registered before participant recruitment).

### 3.2. Caffeine

Since being removed from the World Anti-Doping Agency’s prohibited list in 2004, caffeine has become one of the most widely used ergogenic aids across various sporting events [34]. The ergogenic effects of caffeine have been widely studied in an AB population in long- and short-duration exercise [35]. Guest et al. [36] have published a detailed report on the mechanisms of caffeine and the impact on exercise performance. In short, caffeine has been suggested to enhance myofibrillar calcium availability, optimize exercise metabolism and substrate use, as well as affect the central nervous system, impacting sympathetic drive, motor recruitment, and perception of pain and fatigue [36].

Despite the overwhelming amount of evidence for caffeine as an ergogenic aid in an AB population, evidence for caffeine as an ergogenic aid in a para population remains scarce. In recent years, literature has emerged looking at the effect of caffeine in an SCI population. However, to date, no research has been carried out investigating the ergogenic effects of caffeine in other para populations. There may be altered physiologic and metabolic responses to caffeine in an SCI population, especially in a tetraplegic group, where sympathetic drive is impaired below the lesion level, resulting in impaired catecholamine release [5]. In diseases of the nerves such as Charcot Marie Tooth disease, the literature suggests those affected avoid caffeine in order to manage tremors [37]. However, caffeine may have the potential to counteract the impaired cognitive function in traumatic brain injury [38] through enhancements in cognitive performance during sport participation [39]. Therefore, future research is required in other impairments that are classifiable for para sport.

The current literature on caffeine for an SCI supports the notion that the ergogenic effects of caffeine in this population differ from that found in an AB population, with inconclusive results. While some research indicates no discernible benefit [23,25], others have found similar beneficial effects as in the AB population in both long-duration [32] and short-duration [22,24] exercise. Graham-Paulson et al. [32] utilized multiple doses (2, 4, and 6 mg/kg) taken 45 min before a 20 km handcycling time trial (TT) and found 6 mg/kg to produce greater benefits than 2 mg/kg, which produced greater benefits than 4 mg/kg. All caffeine trials performed better than a placebo. However, this was a case study and, therefore, should be interpreted with caution. Research suggests that 4 mg/kg of caffeine 70 min prior to exercise [5] and 6 mg/kg 60 min before exercise [22] both have ergogenic effects on anaerobic exercise. However, 3 mg/kg taken 60 min prior to exercise [25] and 6 mg/kg taken 90–120 min prior to exercise [23] have no performance-enhancing effects during a graded exercise (VO_2_max) test and anaerobic exercise, respectively. Therefore, optimal timing may lie between 60 and 90 min prior to exercise and optimal doses may be between 4–6 mg/kg, with increased doses required when there is less time between caffeine ingestion and exercise. 

Graham-Paulsen et al. [5] suggested that the pharmacokinetics of caffeine in an SCI population may be altered depending on the level of lesion; therefore, timing and dosage recommendations might need to be adapted on an individual basis. They recommend that tetraplegic athletes require a lower dose to maximize performance, while paraplegic athletes consume caffeine more than 60 min before exercise. Interestingly, some research suggests that handcycling might not benefit from caffeine in a comparable way to cycling, even in an AB population. Graham-Paulson et al. [40] observed no differences in 10 km handcycling TT in AB individuals following the ingestion of 4 mg/kg caffeine 90 min prior to the TT in habitual caffeine users. Therefore, more research is required to determine the effects of caffeine in sport-specific settings. More research is needed to understand the optimal dose and timing in an SCI population. Further, a lack of research exists in other para-athlete populations that are ambulatory but may have different physiological and metabolic responses to caffeine such as brain injury, multiple sclerosis, or degenerative nerve diseases.

### 3.3. Creatine

Research supporting the use of creatine supplementation is vast in exercising individuals to improve strength and power and enhance training adaptations and recovery [41,42]. However, the literature also suggests benefits of creatine supplementation that go beyond exercise performance, such as attenuating age-related losses in muscle mass and bone [43] and neuroprotective effects [44]. A detailed report on the use and safety of creatine can be found in the Position Stand of the International Society of Sports Nutrition [42]. Briefly, creatine supplementation increases phosphocreatine stores, which aids in very short-duration energy production while also acting as an indirect antioxidant and enhancing brain bioenergetics.

Despite the wide array of benefits that creatine supplementation has in both exercise and health in an AB population, the use of creatine in para sport performance remains in its infancy. Studies have shown a direct benefit of creatine on clinical measures in neuromuscular diseases [42], traumatic brain injury [45], and SCI [46]. However, few have investigated the potential ergogenic effect of the supplement in a para-athlete group. Amorim et al. [19] observed increased arm muscle area in a group with SCI following daily ingestion of 3 g of creatine monohydrate paired with an 8-week resistance training program, without increasing 1-RM strength. Conversely, other researchers have observed increased strength following 8 weeks of 10 g and 5 g per day creatine supplementation in adults and children, with muscular dystrophies, without an exercise program [27]. Tarnopolsky & Martin [29] also observed increased strength and decreased muscular fatigue following ~11 days supplementation (10 g/day for 5 days, 5 g/day for another 5–7 days) in people with neurological diseases. Similarly, Jacobs et al. [28] observed increases in peak power output and peak aerobic capacity (i.e., VO_2_peak) with creatine supplementation (20 g per day for seven days) compared to placebo in a cross-over study in an SCI group. In contrast, Perret et al. [26] failed to observe differences between creatine and placebo supplementation (20 g/day for two weeks) on 800 m TT or other variables measured during exercise (i.e., heart rate, lactate, ratings of perceived exertion) in a group of wheelchair athletes. This study differed by using a dosage of 4 × 5 g per day for 6 days, compared to the aforementioned studies that supplemented over a more extended period of time. The International Society for Sports Nutrition [42] recommends a loading phase of 4 × 5 g per day for 5–7 days as sufficient to see benefits, although longer loading periods with increased doses may be necessary to increase brain concentrations of creatine, offset creatine synthesis deficiencies, or influence disease states. This suggests that the protocol used by Perret et al. [26] may have been insufficient to increase phosphocreatine stores adequately to see an improvement in performance. Although the supplementation protocols used by Perret et al. [26] and Jacobs et al. [28] were similar (5 g, 4x/day for 6 and 7 days, respectively), the heterogeneity of the Perret et al. [26] study may have decreased the internal validity of the study compared to the more homogenous group used by Jacobs et al. [28]. There is also a possibility that the minimum amount of time to see meaningful improvements in performance requires 7 days of loading, suggesting the higher end of the recommendations made for an AB population might be considered for athletes with impairments. Further research should determine the optimal dosing strategy for different physical impairments and the impact on different types of Paralympic sports.

### 3.4. Fish Oil

Although the evidence for the use of fish oil is not clear, the Australian Institute of Sport categorizes them as a class “B” supplement, suggesting that research is emerging and the compound is deserving of future research in an athletic population [47]. Omega-3 fatty acid supplementation through the medium of fish oil has been suggested to enhance immune function and decrease inflammatory markers post-exercise, enhancing recovery from exercise [3]. A detailed account of the proposed mechanisms of fish oil in exercising individuals is provided by Mickleborough et al. [48]. In short, fish oils are thought to influence the immune system by acting as a fuel source or through mechanisms related to their role as cell membrane constituents. Fish oil might limit inflammation through inhibiting the formation of pro-inflammatory prostaglandins. Omega-3 fatty acids also appear to have neuroprotective effects by decreasing neuroinflammation and oxidative stress and the activation of cell survival pathways [49].

The single article we found in our literature search suggested that fish oil reduces markers of muscle damage, inflammation, and neutrophil death. Marques and colleagues [30] supplemented eight wheelchair basketball players with 3 g of fish oil daily for 30 days. Before and after supplementation, the athletes’ lipid profile, inflammatory mediators, markers of muscle damage, and neutrophil function were assessed before and after a training session. Following the 30 days of fish oil supplementation markers of muscle damage, inflammatory disturbances, and neutrophil death induced by acute exercise were significantly decreased compared to before supplementation.

Outside of sport, some conditions such as SCI may lead to deficiencies in docosahexaenoic acid. In such a circumstance, supplementation with fish oil may reverse deficiencies, enhancing functional recovery [50]. Other clinical studies have also suggested fish oil to be beneficial in mitigating the increased neuroinflammation and oxidative stress that often accompanies neurological disorders such as multiple sclerosis, SCI, and traumatic brain injury [51]. Further investigation is warranted to indicate if fish oil has beneficial effects on muscle damage and immune markers in an athletic population and if potential benefits correlate with increased performance.

### 3.5. Nitrates

Dietary nitrates, either in the form of sodium nitrate or beetroot juice, increase the body’s access to nitrates, which can then be converted to nitrites and eventually nitric oxide, a potent vasodilator that increases blood flow and oxygen delivery to active tissue during exercise [3]. Nitrate supplementation has also been reported to improve skeletal muscle contractility, mitochondrial efficiency, glucose homeostasis, and respiration [52]. A review by Domínguez et al. [53] provides more insight into the mechanisms of nitrate supplementation. 

Nitrate might have greater performance-enhancing effects on type II muscle fibers, as they demonstrate a shortfall in oxygen delivery relative to demand and reduced contractile efficiency [54,55]. Therefore, nitrates could have greater effect on type II fibers relative to type I through enhancing oxygen delivery via vasodilation and enhancement of contractile efficiency [3,55] This makes it a potentially beneficial supplement for wheelchair athletes or those who propel themselves using their arms, as upper body musculature tends to have a higher percentage of type II muscle fibers [56]. However, the findings of Flueck et al. [20] suggest that neither sodium nitrate nor beetroot juice has beneficial impacts on TT performance in paracyclists with SCI. Yet, although statistical significance was not reached, the beetroot juice and sodium nitrate conditions produced 10 km TT results that were 35 s and 15 s faster than placebo, respectively. As it is not uncommon for competitors to be separated by a time of 3–5 s in international paracyling, this could be a meaningful difference. The lack of statistical findings in this study may be due high variability because of heterogeneity of the participants, with all categories of handcycling (H1-H5) being represented.

Although the use of dietary nitrate has not been explored for exercise performance in other classifiable impairments, clinical data suggest that nitric oxide may have a neuroprotective effect but may also have adverse toxic effects in diseased states. If nitric oxide is produced in an excessive amount, toxicity may result, leading to concern for the diseased/injured brain [57,58]. However, much of such research has been performed in an acute injury or disease state. Very little is known about the role of nitrates and, by extension nitric oxide, in the long-term functioning of those with chronic disease or injury. Therefore, more research is necessary on the clinical outcomes of nitrates in this population. We also suggest further research investigating the effects of beetroot juice for more homogenous para-athlete populations.

### 3.6. Vitamin D

Vitamin D has been well-researched for its role in bone health, and also as an anabolic hormone [59]. As such, adequate vitamin D status (measured as 25(OH)D) is crucial for athletes to optimize their health, training, and performance. In addition to musculoskeletal benefits, vitamin D may also play a role in optimizing immune function and modulating inflammation [60]. Most of the body’s 25(OH)D comes through biosynthesis in the skin following exposure to sunlight, which leaves individuals who reside in areas further away from the equator at risk of being deficient due to insufficient sun exposure. The same can be said for athletes who partake in winter or indoor sports [61]. The mechanisms by which vitamin D may affect performance has been reviewed by Bartoszewska and colleagues [62] and Moran et al. [63].

The literature correlating optimum 25(OH)D status and exercise performance in AB athletes is extensive, with research supporting the use of supramaximal levels for improving performance, including increased aerobic capacity, muscle growth, force, and power production as well as decreased recovery time between exercise bouts [64]. The importance of 25(OH)D in a para-athlete population is also growing, with multiple studies emerging in the current literature search; however, with largely inconclusive results. While some research supports vitamin D supplementation for strength [19] and torque [21] in wheelchair athletes, others [33] have seen no correlation between 25(OH)D levels and performance, despite supplementation to increase 25(OH)D levels. Amorim and colleagues [19] supplemented an SCI population with a 25,000 IU dose of vitamin D every two weeks for 8 weeks (for a total of four doses of 25,000 IU) and found increased strength and arm muscle area after an eight-week strength training intervention compared to a control group. Similarly, Flueck et al. [21] studied the impact of 6000 IU vitamin D daily over 12 weeks in recreationally active individuals with SCI and cerebral palsy who were vitamin D insufficient. Improvements in torque were observed in the non-dominant arm during isometric contraction as well as well as at 180°/s concentrically. However, no differences were observed in peak or mean power or fatigue index measured during a Wingate anaerobic test. Pritchett et al. [33] observed no differences in elite athletes with SCI in 20-m sprint or handgrip strength after supplementing with 15,000–50,000 IU per week for 12–16 weeks. No research has been conducted in other populations of para-athletes to be able to provide evidence-based recommendations.

Despite the lack of evidence in other para-athlete populations, research suggests vitamin D supplementation may be beneficial for health outcomes in those with a physical disability. For example, those with lower-limb amputations may be at risk for decreased bone health in the amputated limb and that adequate supplementation with calcium and vitamin D may help mitigate this risk [65]. Those with severe spastic hemiplegia may have reduced weight-bearing capacity, therefore putting them at risk for low bone mass, for which supplementary vitamin D would be of benefit [66]. Correcting vitamin D deficiency in those with multiple sclerosis may reduce symptoms of the disease [67]. In addition to the impact on bone health, Schnieders and colleagues [68] found that vitamin D deficiency was one of the most critical factors that leads to chronic fatigue in those with a traumatic brain injury. Therefore, the results of the current review suggest that vitamin D may play an essential role in the health of individuals with impairment if they are deficient. However, the research supporting vitamin D supplementation for sports performance in such a population is inconclusive in SCI and non-existent in individuals with other varieties of impairment.

### 3.7. Buffers

The use of different buffering agents to offset the accumulation of H^+^ ions, delaying the onset of muscular fatigue has been researched for over 30 years [69]. Various nutritional strategies have been investigated as potential ways to increase intracellular and extracellular buffering capacity such as beta-alanine, sodium bicarbonate, and sodium citrate [70]. Such buffering agents are of particular interest for sporting events that involve short duration bouts of high intensity exercise, with beta-alanine impacting intracellular buffering capacity and sodium bicarbonate and sodium citrate impacting extracellular buffering [71]. For mechanisms behind buffering agents and exercise performance, the interested reader is directed to Peeling et al. [54] for an in-depth review.

Beta-alanine and sodium bicarbonate have unique impacts on activities of differing lengths, with beta-alanine having small but potentially meaningful effects on activities lasting 30 s to 10 min and sodium bicarbonate being suggested to improve 60 s sprint performance by ~2% [3]. Evidence for the use of sodium citrate as a buffer is limited and, therefore, has not been recommended as a supplement for sport performance [54]. Despite the ergogenic effect of sodium bicarbonate, consumption of this supplement may cause gastrointestinal distress [72] and, thus an individualized approach should be utilized to maximize beneficial effects while minimizing discomfort [3].

Given that muscle mass is a large consumer of lactate in trained individuals during exercise [73], lactate elimination may be impaired in some para-athlete groups due to decreased muscle mass in one or more limbs [74]. This may lead to lactate build-up in lactate-producing muscles. This in turn reduces the strong ion difference within muscle, which contributes to a build-up of [H^+^], leading to increased acidosis [75]. Therefore, buffering agents may have an increased ergogenic effect in this population, including for a broader range of exercise durations. However, the literature on this topic is inconclusive [73]. Furthermore, increased spasticity increases glucose uptake [76] subsequently increasing lactate production even at rest [77]. Despite the array of evidence for various types of buffers in the AB literature, our thorough search of the para literature found a single article, implementing sodium citrate with and without caffeine in elite wheelchair racing athletes [23]. Although sodium citrate effectively increased blood pH and bicarbonate concentrations, it had no effect on 1500 m TT (~3 min) performance. Interestingly, this study saw decreased lactate in the sodium citrate condition compared to the sodium citrate plus caffeine condition, but a trend toward higher lactate levels compared to placebo (*p* = *0*.051). Although only one study, this research suggest sodium citrate has no impact on TT performance in this population. Further research is required to confirm this finding and more research using other varieties of buffers and in para populations is warranted. 

### 3.8. Protein and Amino Acids

Supplementation with protein in the form of powders or bars are common amongst athletes and may be used around a workout to enhance recovery, assist in the accrual of lean mass, or during travel as a form of portable nutrition [3]. Isolated amino acids have also been suggested to maximize muscle protein synthesis, especially the branched chain amino acids (either together or leucine alone). For a review on the efficacy of protein and amino acid supplementation on exercise-related measures, the interested reader is directed to Phillips and Van Loon [78] and Wolfe [79], respectively. In short, supplementation with protein can assist in the accrual of lean body mass when in conjunction with an adequately structured resistance training plan [80], while the use of branched chain amino acids does not promote muscle anabolism [78]. Supplementation with leucine alone has also piqued the interest of the research community due to its role in the activation of the -mammalian target of rapamycin 1 [78]. Research has suggested that, in an AB population, supplementation with leucine in isolation improves power output, ratings of perceived exertion, and time to exhaustion [81].

In a clinical setting, the use of amino acids in the acute stages of brain injury has been well-researched [82,83]. However, the use of amino acid supplementation in chronic brain injury or other physical impairments is absent. The use of an amino acid supplement might be particularly useful to assist in increasing the overall intake of amino acids, which may enhance skeletal muscle growth and development in conditions such as cerebral palsy [84] and muscular dystrophy [85]. Amino acid supplementation, particularly branched chain amino acids (BCAA), might also help combat mental fatigue and “brain fog”, a common side effect of traumatic brain injury [86]. This fatigue can become compounded during physical and mental fatigue brought on by physical exercise. Supplementation with BCAA might increase the ratio of BCAA to free tryptophan, decreasing serotonin production, and potentially delaying mental fatigue and improving exercise performance in this population [87].

Our search yielded a single study using a leucine supplement. Theis et al. [31] supplemented 22 adolescents and young adults with cerebral palsy with 192 mg/kg/day of leucine for 10 weeks. Before and after the intervention, participants were assessed for elbow flexion strength, muscle volume, C-reactive protein, wellbeing, resting energy expenditure, macronutrient oxidation, and body composition. After the 10 weeks, it was found that muscle strength and volume were significantly higher and C-reactive protein was significantly lower in the leucine compared to the control group. No differences in body composition, resting energy expenditure, or macronutrient oxidation were observed. Some wellness measures, including muscle soreness, stress, mood, and general wellbeing, were improved in the leucine group compared to the control group, while others (sleep quality, fatigue) did not differ between the groups. A decrease in C-reactive protein indicates lower levels of inflammation; this may be important for those with sustained neurological conditions, who have elevated inflammation [31]. Therefore, leucine supplementation for those with neurological conditions could have benefits that have not yet been researched.

The paucity of literature surrounding protein and amino acid supplementation in a para-athlete population clearly highlights the need for more research in this area, especially considering the large proportion of this population who currently use protein and amino acid supplements [4,7]. Future research should investigate the efficiency of different protein and amino acid supplements for both physical and cognitive performance (decreasing cognitive fatigue), as well as highlight optimal dosing strategies. 

### 3.9. Future Recommendations

Despite the use of dietary supplements in para-athletes being widespread [4], the evidence supporting the use of supplements in this population remains scarce. While consensus statements have been produced on evidence-based supplements [3,36,42] in an AB population, the research remains lacking in a para-athlete population. Due to different physiological or metabolic responses concerning their impairment or the nature of their sport, sport supplements might have differing effects in a para group compared to their AB peers. Therefore, recommendations from the AB literature should not be extrapolated to individuals with a physical impairment without caution. This review also revealed that research in a para population is almost exclusively in SCI, suggesting that future research should investigate the use of supplements in different classifiable impairments (cerebral palsy, neurodegenerative conditions, brain injury, amputation, etc.).

Future research should also focus on broadening the scope of supplements with an evidence-base for or against. At the time of this review, only seven types of supplements had been investigated, with many having a single study investigating the effects. As such, an excellent opportunity for research exists in this field, especially, in the following domains:

Cannabidiol: Cannabinoids have been used historically for a variety of clinical populations but were not backed by scientific evidence until the early 2000’s [88]. Cannabidiol (CBD) is a non-psychoactive cannabinoid that has been suggested to provide potential therapeutic health benefits without the adverse side effects associated with other cannabinoids [88]. Although the use of CBD for health outcomes is emerging [89], the use of CBD in a sporting population is non-existent, likely due to concerns over contamination, as all forms of cannabinoids except CBD are considered a banned substance in competition by the World Anti-Doping Agency [90]. However, in a para-athlete population, CBD has the potential to increase performance through multiple indirect pathways, such as managing chronic pain associated with conditions such as SCI, multiple sclerosis, and amputation [91], or to manage spasticity that might be present in conditions such as multiple sclerosis, brain injury, or cerebral palsy [92]. Future research should focus on the impact of CBD on the overall quality of life in this population and if the therapeutic effects have the potential to increase the quality of training, therefore improving performance.

## 4. Conclusions

Despite the increase in sport participation in individuals with a physical impairment, the literature is severely lacking for dietary supplements to increase athletic performance. Due to crucial physiological and metabolic differences, recommendations for dietary supplements should not be provided to a para-athlete population based on the AB literature. It is essential to understand that the scope of classifiable physical impairments span much more extensively than individuals with SCI, and therefore more research in populations with different physical impairments is required to develop recommendations on evidence-based dietary supplements for this population. Supplement use in the populations addressed in this review is inconclusive, and thus individualized recommendations should be considered for those interested in consuming dietary supplements. 

As with any athletic population choosing to consume supplements, the athlete should be aware of potential risks of supplementing and be informed of the anti-doping regulations of their sport.

## Figures and Tables

**Table 1:** Studies involving dietary supplements in para-athletes.

Reference	Study Design	Population (n)	Supplement	Dosing Strategy	Outcome Measures	Results
Flueck et al. 2019	DB, RCT, crossover	AB (14) and paracyclists with SCI (8)	-Beetroot juice -Sodium nitrate	-6 mmol nitrate 3 h before TT	-PO -10 km TT -HR -BP -Blood lactate -RPE -PO: VO_2_ ratio	-Nitrate levels were higher in beetroot compared to placebo. SCI population showed greater performance than AB. Nitrite levels were higher in AB than SCI group. AB group saw improved PO:VO_2_ ratio in some km
Flueck et al. 2014	DB, RCT, crossover	-Elite wheelchair racing athletes (9)	-Caffeine -Sodium citrate -Sodium citrate +caffeine	-Caffeine: 6 mg/kg -Sodium Citrate: 0.5 g/kg -Caffeine + sodium citrate (as above) 90–120 min before exercise tests	-1500 m TT -HR_max_ -HR_average_ -Blood lactate -Blood ph -Blood bicarbonate	-Blood ph was higher at rest after sodium citrate and sodium citrate +caffeine conditions -Blood bicarbonate was higher after the TT in the sodium citrate and sodium citrate +caffeine conditions -Maximal lactate concentrations were higher in caffeine and caffeine +sodium citrate conditions
Graham-Paulson et al. 2016	DB, RCT, crossover	Wheelchair rugby players (12)	-Caffeine	-4 mg/kg 70 min before exercise tests	-Subjective feeling measures -3 × 20 m sprint -4 × 4 min maximal push -RPE	-Caffeine improved 20 m sprint and improved the first 4-min push effort, but not the others. The caffeine condition also showed decreased RPE scores
Graham-Paulson et al. 2018	Case Study; single blind, randomized, placebo controlled, repeated measures	SCI (1)	-Caffeine	-2 mg/kg -4 mg/kg -6 mg/kg 45 min before exercise tests	-20 km TT -Blood glucose -Blood lactate -HR -RPE	-TT performance: 6 mg/kg > 2 mg/kg > 4 mg/kg > placebo -Blood lactate was highest in the 6 mg/kg trial -Average HR was highest under 6 mg/kg, then 2 mg/kg, then 4 mg/kg
Klimešová et al. 2017	DB, RCT, crossover	Wheelchair rugby players (7)	-Caffeine	-3 mg/kg 60 min before exercise	-VO_2_peak -RPE -PO_max_	-Caffeine did not improve any of the outcomes measured
Flueck et al. 2015	DB, RCT, crossover	AB (17), paraplegics (10), tetraplegics (7)	-Caffeine	6 mg/kg 60 min before exercise tests	-PO -Fatigue Index -Plasma catecholamines -Plasma caffeine -Lactate	-Paraplegic group showed higher 30 s and 1 min PO in the caffeine condition compared to placebo -Tetraplegics had significantly greater plasma caffeine concentration 1 h after supplementation compared to AB and significantly more lactate after exercise in the caffeine condition compared to placebo
Amorim et al. 2018	DB, RCT	Adults with SCI (14)	-Creatine -Vit D	-Creatine: 3 g/day for 8 weeks -Vit D: 25,000 IU Vit D every 2 weeks for 8 weeks	-anthropometrics -arm muscle area -grip strength -seated medicine ball throw -Muscular strength (1 RM) -wheelchair slalom test -Plasma Vit D	-Creatine increased Arm muscle area -Positive correlation between Vit D status and Pec Deck 1 RM
Perret et al. 2006	DB, RCT, crossover	Wheelchair athletes (6)	-Creatine	-4 × 5 g/day for 6 days	-800 m TT -HR -RPE -Blood lactate	-Creatine had no impact on the outcomes measures
Walter et al. 2000	DB, RCT, crossover	Patients with muscular dystrophy (36)	-Creatine	-10 g/day for adults, 5 g/day for children for 8 weeks	-Muscular strength	-Creatine increased strength
Jacobs et al. 2002	DB, RCT, crossover	SCI (16)	-Creatine (chronic)	-5 g 4x/day for 7 days	-VO_2_ -HR -RPE -PO	-Creatine group reached significantly greater values of VO_2_, VCO_2_, tidal volume, and PO
Tarnopolsky et al. 1999	Single-blind, placebo controlled	Neurological conditions (102 over 2 studies)	-Creatine (chronic)	-10 g/day for 5 days, followed by 5 g per for 5 to 7 days	-ankle dorsiflexion strength -ankle dorsiflexion fatigue -knee extension strength -grip strength	-Creatine group saw improvements in ankle dorsiflexion strength and fatigue, grip strength, and knee extension strength compared to the placebo
Marques et al. 2016	Cross-sectional	Wheelchair basketball players with SCI (8)	-Fish oil	-3 g fish oil (1500 mg DHA, 300 mg EPA) per day for 30 days	-Muscle damage -Inflammation	-Fish oil reduced markers of muscle damage, inflammatory disturbances, and neutrophil death induced by acute exercise
Flueck et al. 2016	DB	Wheelchair athletes (20)	-Vit D	-6000 IU daily for 12 weeks	-elbow flexion torque Blood lactate -HR_max_ -RPE -PO_max_ -PO_average_ -fatigue index -plasma Vit D -plasma calcium -upper extremity function	-Supplementation increased plasma Vit D and increased torque in non-dominant arm at 0 and 180°/s. There was also a positive correlation between 60°/s torque and Vit D status in non-dominant arm
Pritchett et al. 2019	Single-arm	Spinal cord impairment- SCI, spina bifida, cauda equina (34)	-Vit D	-15,000–50,000 IU per week for 12–16 weeks	-3 × 20 m sprint -grip strength	-Supplementation increased serum Vit D levels -Vit D did not improve any of the performance measures
Theis et al. 2020	DB, RCT	Adolescents and young adults with cerebral palsy (21)	-Leucine	-192 mg/kg for 10 weeks	-elbow Flexor Strength -muscle volume -CRP -wellbeing -resting energy expenditure -body composition	-Muscle strength, volume, and CRP increased in leucine group compared to control -Measures of wellbeing (stress, muscle soreness, mood, and general wellbeing) improved in leucine group compared to control

SCI = spinal cord injury; DB = double blind; VO_2_ = oxygen consumption; HR = heart rate; TT = time trial; BP = blood pressure; RPE = ratings of perceived exertion; AB = able-bodied; PO = power output; VCO_2_ = expired carbon dioxide; RCT = randomized controlled trial; CRP = C-reactive Protein.

**Table 2:** Risk of Bias for Selected Studies.

Study		Risk of Bias Domain					
	Randomization Process	Period or Carry-Over Effect	Deviation from Intended Intervention	Missing Outcome Data	Measurement of Outcome	Selection of Reported Results	Overall Risk of Bias
Beetroot Juice							
Flueck et al. 2019	Low	Low	Low	Low	Low	Low	Low
Caffeine							
Flueck et al. 2014	Low	Low	Low	Low	Low	Some concerns	Some concerns
Graham-Paulson et al. 2016	Low	Low	Low	Low	Low	Some concerns	Some concerns
Flueck et al. 2015	Low	Low	Low	Low	Low	Some concerns	Some concerns
Klimešová et al. 2017	Low	Low	Low	Low	Low	Some concerns	Some concerns
Creatine							
* Amorim et al. 2018	Low	N/A	Low	Low	Low	Low	Low
Perret et al. 2006	Low	Low	Low	Low	Low	Some concerns	Some concerns
Walter et al. 2000	Low	Low	Low	Low	Low	Some concerns	Some concerns
Jacobs et al. 2002	Low	Low	Low	Low	Low	Some concerns	Some concerns
Tarnopolsky et al. 1999	Low	N/A	N/A	Low	Low	Some concerns	Some concerns
Fish Oil							
Marques et al. 2016	High	Low	Low	Low	Low	Some concerns	High
Vitamin D							
Flueck et al. 2016	High	N/A	Low	Low	Low	Low	High
Pritchett et al. 2019	High	Low	Low	Low	Low	Some concerns	Some concerns
Protein and Amino Acids							
Theis et al. 2020	Low	N/A	Low	Low	Low	Low	Low

* Amorim et al. (2018) also investigated vitamin D.

## Data Availability

Not applicable.
